# Spontaneous Pneumothorax Diagnosed As Birt-Hogg-Dubé Syndrome: A Report of a Rare Case

**DOI:** 10.7759/cureus.79875

**Published:** 2025-03-01

**Authors:** Ishan A Sane, Jessica R Gupte, Himanshu Pophale, Pankaj Magar, Unmesha Pawar

**Affiliations:** 1 College of Medicine, Smt. Kashibai Navale Medical College and General Hospital, Pune, IND; 2 Respiratory Medicine, Smt. Kashibai Navale Medical College and General Hospital, Pune, IND

**Keywords:** birt-hogg-dubé syndrome (bhds), diagnostic genetic testing, flcn gene mutation, pneumothorax (ptx), pulmonary cysts

## Abstract

The aim of this case report is to highlight the rare occurrence of spontaneous pneumothorax as the initial presentation of Birt-Hogg-Dubé (BHD) syndrome, its diagnostic modalities, manifestations, and treatment approach. A spontaneous pneumothorax occurs when air enters the pleural space in the absence of trauma. Primary spontaneous pneumothorax, which occurs in the absence of any underlying cause, is generally due to the rupture of subpleural emphysematous blebs. Mutations in a tumour-suppressor gene (FLCN gene) result in BHD syndrome, an autosomal dominant condition, which is usually characterized by the formation of pulmonary cysts, skin fibrofolliculomas, and renal tumours.

We report a case of a 38-year-old male, a non-smoker, who presented with complaints of right-sided chest pain, shortness of breath, and dry cough. Clinical examination showed reduced chest movements, hyper-resonance notes, and reduced breath sound on the right hemithorax. Chest X-ray showed pneumothorax on the right side, and high-resolution computed tomography (HRCT) of the thorax showed multiple thin-walled cysts in bilateral lungs. Whole genomic sequencing identified mutations in the FLCN gene, confirming the diagnosis of BHD syndrome. Management included intercostal drainage tube insertion for pneumothorax, high-flow oxygen therapy, and symptomatic care. Post-discharge recommendations included pneumococcal and annual influenza vaccinations, pulmonary function monitoring, renal tumour screening, and genetic counselling for family members.

This case highlights that not all cases of BHD syndrome present with the classical triad of pulmonary, skin, and kidney abnormalities. Some cases may manifest solely with pulmonary features, such as spontaneous pneumothorax. Pulmonary cyst rupture is a hallmark of BHD syndrome and often leads to recurrent pneumothorax. Early diagnosis and genetic testing are critical for optimal management and prognosis.

This case emphasizes the variability in BHD syndrome presentation and underscores the importance of comprehensive evaluation, genetic testing, and long-term surveillance to manage potential complications. Genetic counselling of family members is essential to identify at-risk individuals and ensure timely interventions.

## Introduction

Birt-Hogg-Dubé (BHD) syndrome, a rare genetic disorder with autosomal dominant inheritance, was initially described in 1977. This condition is characterized by the presence of lung cysts, spontaneous pneumothorax, renal cancer, and skin fibrofolliculomas [1]. The prevalence of BHD syndrome is approximately 2 cases per 1,000,000 individuals [2]. The protein associated with BHD syndrome is extensively distributed across various tissues, including the kidneys, lungs, and skin [3]. The precise incidence of this syndrome remains unknown. This inherited condition results from mutations in the folliculin (FLCN) gene on chromosome 17p11.2, which codes for the folliculin protein. This protein plays a vital role in cellular signaling pathways, such as the mechanistic target of the rapamycin (mTOR) pathway, which is involved in cellular growth and multiplication [4]. The diverse clinical manifestations of BHD syndrome often lead to its underdiagnosis. Key diagnostic indicators include recurring pneumothorax, cysts or bullae visible on the high-resolution computed tomography (HRCT) chest, multiple skin lesions, kidney cysts or lesions, and/or a family history of pneumothorax [5].

The present report showcases a patient with BHD syndrome, with mutations in the folliculin gene, who presented with spontaneous pneumothorax and multiple cysts in the lungs.

## Case presentation

**Figure 1:**
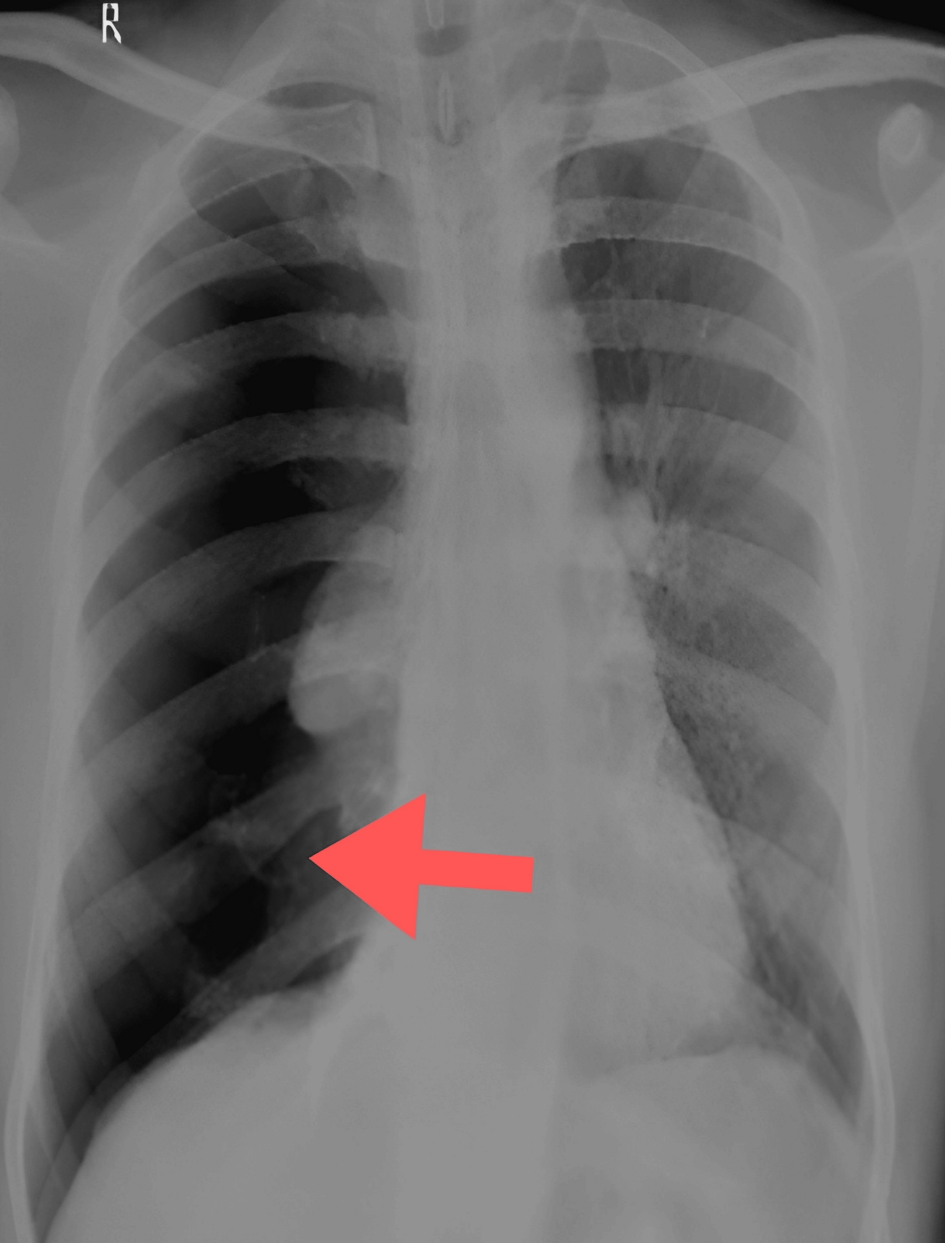
Chest X-ray showing right-sided pneumothorax (arrow).

**Figure 2:**
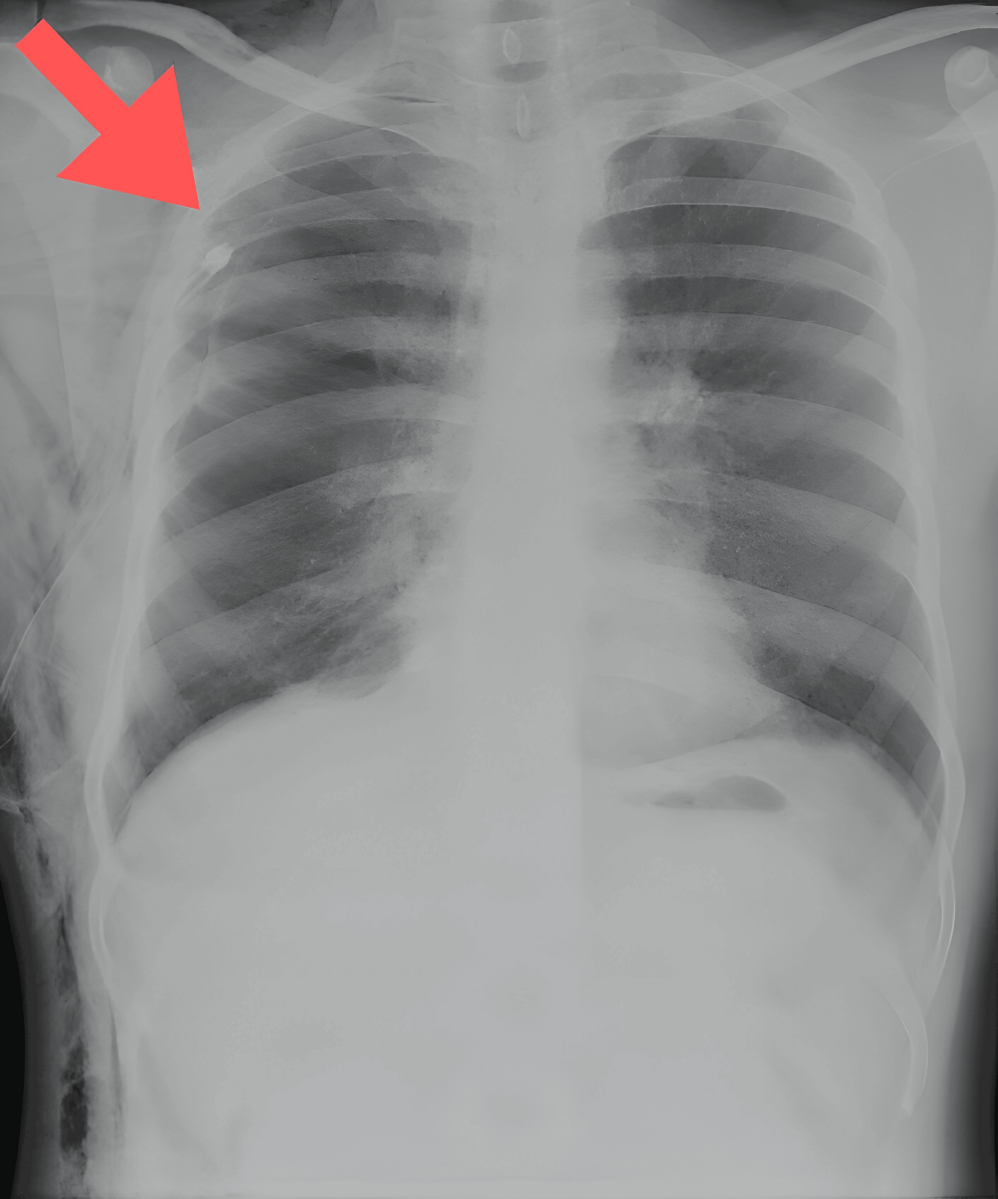
Chest X-ray post-intercostal drainage tube insertion (arrow indicating the intercostal drainage tube).

A 38-year-old male came to the casualty with complaints of right-sided chest pain, shortness of breath for one week, and cough on and off for the last one year, which was occasionally productive, for which the patient took medications potentially indicating underlying lung involvement. The patient had no relevant family history, prior similar episodes in the past, addictions, comorbidities, allergies, or history of chest trauma or recent air travel. The patient had a history of COVID-19 infection in 2021, for which he did not require hospitalization. Clinical examination revealed reduced chest movements, hyper-resonant percussion notes, and reduced breath sounds on the right hemithorax. Chest X-ray showed pneumothorax on the right side, which was managed by inserting a chest tube in the pleural space following aseptic precautions (Figures 1-2).

On admission, baseline laboratory investigations were within normal range. Sputum studies showed few epithelial cells, and Gram-positive cocci were seen. No organism growth was observed in culture studies. No fungal elements were seen. No acid-fast bacilli were seen on the Ziehl-Neelsen (ZN) stain. Bronchial washings were also inconclusive. There were no other features of tuberous sclerosis or a history of similar disease in the family. Serum immunoglobulin E (IgE) was within normal limits, and antinuclear antibody (ANA) by immunoflorescence method was negative. Alpha-1 antitrypsin values were within normal limits.

Initially, oral and intravenous antibiotics (intravenous amoxicillin-clavulanate 2 g) were administered, and daily intercostal drainage tube care was provided. The patient was started on high-flow oxygen, and the intercostal drainage tube was connected to Sinapi’s chest drain (Sinapi Biomedical, Stellenbosch, South Africa).

**Figure 3:**
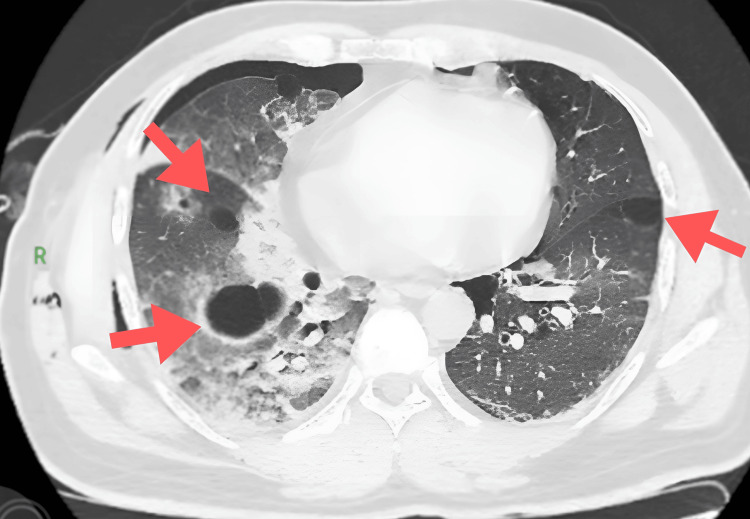
High-resolution computed tomography (HRCT) thorax showing lung cysts in a patient with Birt-Hogg-Dubé (BHD) syndrome (arrows indicating multiple pulmonary cysts in the lung parenchyma).

**Figure 4:**
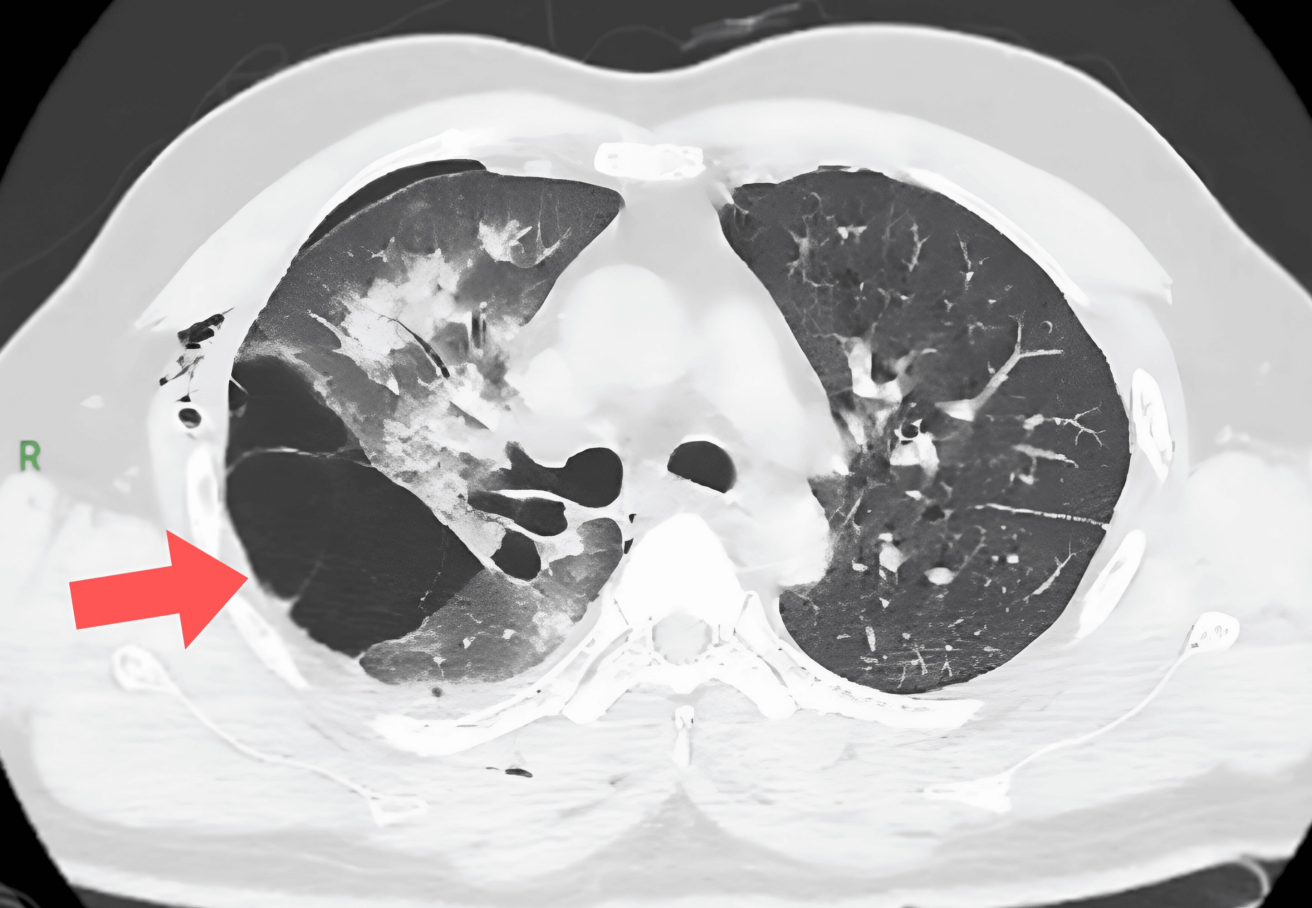
High-resolution computed tomography (HRCT) thorax showing lung cysts in a patient with Birt-Hogg-Dubé (BHD) syndrome (arrow indicating a large pulmonary cyst in the lung parenchyma).

An abdominal ultrasound revealed no tumors. HRCT of the thorax revealed interstitial septal thickening with areas of ground-glass opacities that coalesced with patchy consolidation and an air bronchogram within the right lung. Additionally, HRCT showed multiple well-defined thin-walled cysts of varying sizes in bilateral lungs, primarily in subpleural locations, which tend to be located at the basilar and mediastinal regions of the lungs. There was no evidence of fibrosis or honeycombing (Figures 3-4).

Genetic testing was performed using targeted gene sequencing, which identified a heterozygous single-base pair deletion in exon 11 of the FLCN gene on chromosome 17, confirming a mutation in the genomic sequence (Table 1). The genetic testing of the patient was classified based on the recommendations of the American College of Medical Genetics and Genomics (ACMG) guidelines, with the detected variant categorized as pathogenic (PVS1, PM2, PP5) [6]. Based on these findings, a diagnosis of BHD syndrome was established.

**Table 1:** Genetic testing report of the patient showing a mutation in the FLCN gene.

Gene (transcript)	Location	Variant	Zygosity	Disease (OMIM)	Inheritance	Classification
FLCN (-) (ENST00000285071.9)	Exon 11	c1285del (p.His429ThrfsTer39)	Heterozygous	Primary spontaneous pneumothorax (OMIM#173600)	Autosomal dominant	Pathogenic (PSV1, PM2, PP5)

The patient was managed symptomatically, with pneumothorax relieved by intercostal drainage tube insertion. The patient showed significant improvement as symptoms gradually subsided. The patient was referred to the surgery department, which advised the patient to undergo video-assisted thoracic surgery (VATS) and mechanical pleurodesis in case of recurrent pneumothoraces. After three days of hospitalization, the patient was discharged. Pneumococcal and annual influenza vaccinations, along with periodic pulmonary function testing, were advised to the patient. Annual screening for renal tumors with MRI scans and genetic counseling of the family members were recommended. In case of any skin lesions that may occur in the future, the patient was advised to get a dermatological consultation.

## Discussion

In our case, pneumothorax was resolved by the insertion of an intercostal drainage tube, after which the patient became stable. High-flow oxygen was also given, along with daily intercostal drainage tube care. The diagnosis of BHD syndrome was made based on radiological investigations and confirmed by genetic testing, which identified a mutation in the FLCN gene. BHD syndrome is an uncommon genetic condition inherited in an autosomal dominant manner. It is characterized by three main clinical features: fibrofolliculomas on the skin, tumors in the kidneys, and multiple cysts in the lungs [1].

The primary causes of death and illness in BHD syndrome are directly linked to internal manifestations of the disease, such as pneumothorax or renal cell carcinoma [3]. Patients are recommended to avoid cigarette smoking, high ambient pressures, and radiation exposure. Immediate family members should undergo genetic testing to identify the presence of the family-specific pathogenic variant [5]. Typically, BHD syndrome is diagnosed when individuals are in their 40s or 50s. The condition results from mutations in the FLCN gene located at the 17p11.2 locus, which encodes the protein folliculin [7].

In our case, the patient experienced only spontaneous pneumothorax, without skin or kidney complications, a common but not universal presentation of BHD syndrome. Spontaneous pneumothorax can be the initial presentation of BHD syndrome and may be the sole manifestation in some cases. The majority of BHD syndrome patients show radiological evidence of lung cysts and/or bullae. The most comprehensive and extensive study of individuals and families with BHD syndrome found that 89% of patients had multiple pulmonary cysts [8].

In BHD syndrome, spontaneous pneumothorax occurs due to the rupture of lung cysts. Not all patients exhibit all three manifestations (skin, lung, and kidney); rather, some may only have multiple pulmonary cysts without other features [9]. However, since BHD syndrome increases the risk of renal tumors, screening of affected patients should be considered [10]. Due to the brief hospital stay in this case, the long-term risk of renal tumors could not be evaluated, but the patient was counseled and advised to have routine MRI scans in the future to detect any evidence of renal tumors.

## Conclusions

This case highlights the key clinical features of the rare genetic disorder BHD syndrome, emphasizing its variable presentation and the diagnostic approaches required for confirmation. Notably, this syndrome does not always manifest as the classic triad of pulmonary, renal, and cutaneous involvement; instead, many cases present only with pulmonary symptoms, like spontaneous pneumothorax, highlighting the need for careful clinical consideration to ensure timely diagnosis.

Optimal management involves symptomatic treatment, comprehensive patient counseling regarding potential future complications, and regular screening to monitor disease progression. Long-term follow-up is essential for effective disease management. Early diagnosis, coupled with genetic counseling for family members, plays a crucial role in improving prognosis and facilitating timely interventions for at-risk individuals.

## References

[1] Yukawa T, Fukazawa T, Yoshida M (2016). A case of Birt-Hogg-Dubé (BHD) syndrome harboring a novel folliculin (FLCN) gene mutation. Am J Case Rep.

[2] Muller ME, Daccord C, Taffé P, Lazor R (2021). Prevalence of Birt-Hogg-Dubé syndrome determined through epidemiological data on spontaneous pneumothorax and Bayes theorem. Front Med (Lausanne).

[3] Rashmi K, Arbind D, Patanjali C, Kunal G (2012). Brit Hogg Dube syndrome - a rare disease entity with review of literature. J Clin Case Rep.

[4] Leivaditis V, Papatriantafyllou A, Koletsis E (2023). A rare case of spontaneous pneumothorax recurrence 30 years after surgery in a patient with Birt-Hogg-Dube syndrome: case presentation and short review of the literature. Acta Inform Med.

[5] Varnana Suresh AT, Gowtham Kumar, Keny S (2022). Birt-Hogg-Dubé syndrome: a case report. Int J Sci Res.

[6] Richards S, Aziz N, Bale S (2015). Standards and guidelines for the interpretation of sequence variants: a joint consensus recommendation of the American College of Medical Genetics and Genomics and the Association for Molecular Pathology. Genet Med.

[7] Ptak B, Wąsik G (2021). Birt-Hogg-Dubé syndrome. Dermatol Rev/Przegl Dermatol.

[8] Rehman HU (2012). Birt-Hogg-Dubé syndrome: report of a new mutation. Can Respir J.

[9] Özer EA, Pampal HK, Dakak M (2012). Multiple lung cysts and Birt-Hogg-Dube syndrome: management of anaesthesia and surgery. Gulhane Med J.

[10] Andrews SN, Khrisnadas R, Berrisford RG, Froeschle PO (2007). Pneumothorax and Birt-Hogg-Dube syndrome: diagnostic and therapeutic aspects. Resp Med Thorac Surg.

